# Supplementary material for: Culture Isolate of Rickettsia felis from a Tick
Source: Int J Environ Res Public Health. 2022 Apr 4;19(7):4321. doi: 10.3390/ijerph19074321 (PMC8998211; doi:10.3390/ijerph19074321)
Supplement: Supplementary file 1 [file ijerph-19-04321-s001.zip › ijerph-1596003-supplementary.pdf]

Figure S1.

Centrifugation tubes with *R. felis* isolate Danube purified from XTC-2 cells by isopycnic density gradient ultracentrifugation. Rickettsiae were purified from twenty cultivation flasks with a growth area of 75 cm^2 of heavily infected cells. The thin white arrow points to the host cell debris at the 30% renografin layer and the thick white arrow points to a band of viable rickettsiae, sedimented between the 30%–36% renografin interface.

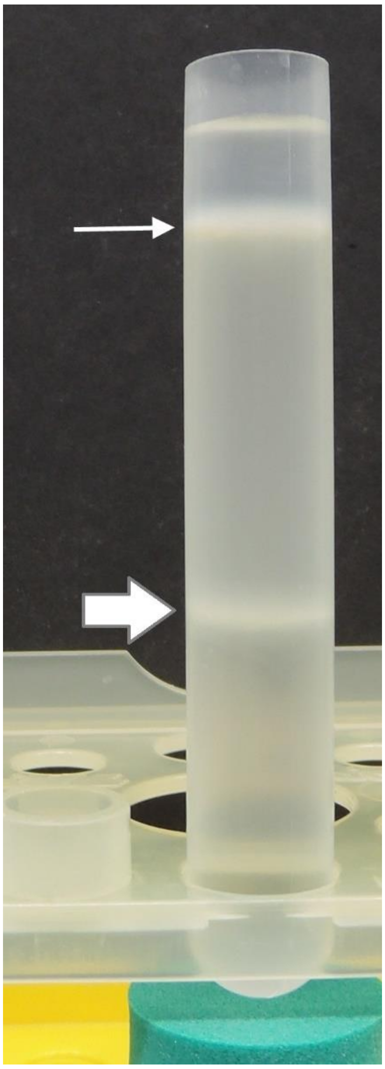

Figure S2.

PCR confirmation of the absence of *R. helvetica* contamination in the purified isolate Danube. Agarose gel electrophoresis of PCR products of the gene coding 23S rRNA, amplified with *R. helvetica*-specific primer pair Rh16f and Rh356r. M: molecular marker; 1: *R. helvetica* C9P9; 2: *R. helvetica* IR16; 3: *R. felis* Danube; 4: negative control.

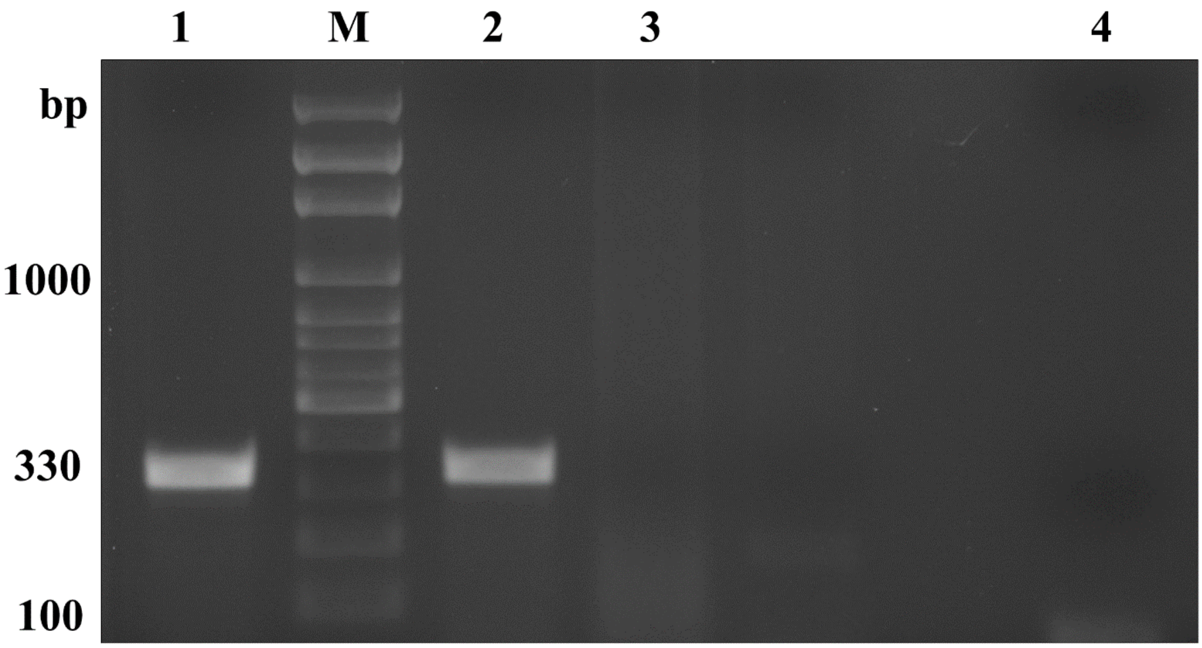

Table S1. Molecular detection of *R. felis* in ticks.

Year	Tick species	Life stage	Origin	Country	Reference
2003	*Haemaphysalis flava*<br>*Haemaphysalis kitasatoe*<br>*Ixodes ovatus*	Adult and nymph	Questing	Japan	Ishikura M., Ando S., Shinagawa Y., *et al. Microbiol Immunol* 47(11):823-832 [24]
2006	*Amblyomma cajennense*<br>*Rhipicephalus sanguineus*	Adult	Collected from *Equus caballus* and *Canis familiaris*	Brazil	Cardoso L.D., Freitas R.N., Mafra C.L., *et al. Cad Saude Publica* 22(3):495-501 [14]
2006	*Carios capensis*	N/A*	Collected from nests of *Pelecanus occidentalis*	The United States	Reeves W.K., Loftis A.D., Sanders F., *et al. Exp Appl Acarol* 39(3-4):321-329 [43]
2006	*Haemaphysalis sulcata*	Adult	Collected from *Ovis aries* and *Capra hircus*	Croatia	Duh D., Punda-Polic V., Trilar T., *et al. Ann N Y Acad Sci* 1078:347-351 [25]
2007	*Ixodes granulatus*	N/A*	Collected from *Suncus murinus*	Taiwan	Tsui P.Y., Tsai K.H., Weng M.H., *et al. Am J Trop Med Hyg* 77(5):883-890 [33]
2008	*Rhipicephalus sanguineus*	Adult male	Collected from *Canis familiaris*	Brazil	Oliveira K.A., Oliveira L.S., Dias C.C., *et al. Mem Inst Oswaldo Cruz* 103(2):191-194 [15]
2009	*Rhipicephalus sanguineus*	Adult	Feeding on an animal (species not specified)	Spain	Toledo A., Olmeda A.S., Escudero R., *et al. Am J Trop Med Hyg* 81(1):67-74 [16]
2009	*Ixodes ricinus*	N/A*	N/A	Germany	Dobler G. and Wölfel R. *Dtsch Arztebl Int* 106(20):348-354 [35]
2012	*Rhipicephalus bursa*	Adult male	Removed from a human	Turkey	Gargili A., Palomar A.M., Midilli K., *et al. Vector Borne Zoonotic Dis* 12(11):938-941 [22]
2012	*Amblyomma maculatum*	Adult female and male	Removed from a human and *Canis familiaris*	The United States	Jiang J., Stromdahl E.Y., and Richards A.L. *Vector Borne Zoonotic Dis* 12(3):175-182 [29]
2013	*Rhipicephalus sanguineus*	N/A*	Collected from *Canis familiaris*	Chile	Abarca K., López J., Acosta-Jamett G., *et al. Vector Borne Zoonotic Dis* 13(8):607-609 [17]
2014	*Rhipicephalus sanguineus*	N/A*	Collected from *Canis familiaris*	China	Zhang J., Lu G., Kelly P., *et al. BMC Infect Dis* 14:682 [18]
2015	*Amblyomma humerale*	Nymph	Collected from *Didelphis* sp.	Brazil	Soares H.S., Barbieri A.R., Martins T.F., *et al. Exp Appl Acarol* 65(1):125-140 [30]

2017	*Haemaphysalis leporispalustris*	Larvae and adult female	Questing larvae and female tick collected from *Lepus californicus*	The United States	Roth T., Lane R.S., and Foley J. *J Med Entomol* 54(2):492-495 [26]
2017	*Haemaphysalis* spp. *Rhipicephalus microplus*	N/A*	Collected from *Canis familiaris* and *Bos taurus*	Malaysia	Kho K.L., Koh F.X., Hasan L.I., *et al. Emerg Microbes Infect* 6(4):e18 [27]
2017	*Amblyomma sculptum*	Adult female	Questing	Brazil	Bitencourth K., Amorim M., de Oliveira S.V., *et al. Med Vet Entomol* 31(4):427-437 [31]
2018	*Haemaphysalis bancrofti*	Adult	Collected from *Equus caballus*	Australia	Chalada M.J., Stenos J., Vincent G., *et al. Vector Borne Zoonotic Dis* 18(3):151-163 [28]
2018	*Rhipicephalus turanicus*	Adult	Collected from *Ovis aries*	Italy	Raele D.A., Galante D., Pugliese N., *et al. Microbiologyopen* 7(1) [23]
2019	*Amblyomma ovale*	Adult male	Collected from *Canis familiaris*	Brazil	Bitencourth K., Amorim M., de Oliveira S.V., *et al. Med Vet Emtomol* 33(2):256-268 [32]
2019	*Ixodes hexagonus*	Adult	Collected from *Erinaceus europaeus* and *Vulpes vulpes*	Italy	Pascucci I., Di Domenico M., Curini V., *et al. Microorganisms* 7(12) [34]
2019	*Ixodes ricinus*	Nymph	Questing	France	Lejal E., Marsot M., Chalvet-Monfray K., *et al. Parasit Vectors* 12(1):551 [36]
2019	*Ixodes ricinus*	Adult	Questing; salivary glands	France	Lejal E., Moutailler S., Šimo L., *et al. Parasit Vectors* 12(1):152 [37]
2019	*Ixodes ricinus*	N/A*	Questing	Spain	Remesar S., Díaz P., Portillo A., *et al. Exp Appl Acarol* 79(2):267-278 [38]
2020	*Rhipicephalus sanguineus*	Adult	Collected from *Canis familiaris*	Brazil	Campos S.D.E., Cunha N.C.D., Machado C.S.C., *et al. Rev Bras Parasitol Vet* 29(4):e014220 [20]
2020	*Dermacentor nitens* *Rhipicephalus sanguineus*	N/A*	Collected from *Equus caballus* and *Canis familiaris*	Brazil	de Oliveira J.C.P., Reckziegel G.H., Ramos C.A.D., *et al. Exp Appl Acarol* 81(2):255-264 [21]
2020	*Rhipicephalus sanguineus*	Adult male	Collected from *Canis familiaris*	The Philippines	Nguyen V.L., Colella V., Greco G., *et al. Parasit Vectors* 13(1):420 [19]
2020	*Dermacentor nitens*	Adult female	Collected from *Equus caballus*	Cuba	Díaz-Sánchez A.A., Chilton N.B., Roblejo-Arias L., *et al. Med Vet Entomol* 35(2):207-212 [41]
2021	*Ixodes ricinus*	Nymph	Removed from *Turdus merula*	Romania	Borşan S.D., Ionică A.M., Galon C., *et al. Front Microbiol* 12: 645002 [39]

2021	*Ixodes ricinus*	Adult female	Removed from a human	Serbia	Banović P., Díaz-Sánchez A.A., Galon C., *et al.* *Ticks Tick Borne Dis* 12(2):101609 [40]
2021	*Dermacentor variabilis*	Adult	Removed from a companion animal	The United States	Stanley H. and Rhodes D.V.L. *Vet Sci* 8(3) [42]

Table S2. Oligonucleotides used in the study for conventional PCR and sequencing of the *R. felis* strain Danube.

Oligonucleotide name	Target gene	Sequence (5'→ 3')	Amplicon size (bp)*	Reference
fD1	*rrs*	AGA GTT TGA TCC TGG CTC AG	1462	Roux V. and Raoult, D. (1995) *Res Microbiol* 146(5):385-396 [72]
rp2		ACG GCT ACC TTG TTA CGA CTT		
fD1		AGA GTT TGA TCC TGG CTC AG	753	Al Masalma M., Armougom F., Scheld W.M., *et al.* (2009) *Clin Infect Dis* 48(9):1169-1178 [73]
800r		CTA CCA GGG TAT CTA AT		
D328AAEf	*sca*4	GAC CGT GAT TTA GCT GAA CA	1033	Sekeyova Z., Roux V., and Raoult D. (2001) *Int J Sys Evol Microbiol* 51(4):1353-1360 [74]
D1357AAEr		TAA GAA TTG CNG TAG CTG CT		
D767f		CGA TGG TAG CAT TAA AAG CT	633	
D1390r		CTT GCT TTT CAG CAA TAT CAC		
D1204AAEf		TCG CAA CAA GTG AAT CCA AA	880	
D2077AAEr		CAA TGT CTG CTT TAT CTT G		
Rr17k.1p	*htr*A	TTT ACA AAA TTC TAA AAA CCA T	541	Ishikura M., Ando S., Shinagawa Y., *et al.* (2003) *Microbiol Immunol* 47(11)823-832 [24]
Rr17k.539n		TCA ATT CAC AAC TTG CCA TT		
CS-78	*glt*A	GCA AGT ATC GGT GAG GAT GTA AT	401	Labruna M.B., Whitworth T., Horta M.C., *et al.* (2004) *J Clin Microbiol* 42(1):90-98 [75]
CS-323		GCT TCC TTA AAA TTC AAT AAA TCA GGA T		
CS877f		GGG GAC CTG CTC ACG GCG G	477	Roux V., Rydkina E., Eremeeva M., *et al.* (1997) *Int J Syst Bacteriol* 47(2):252-261 [76]
CS1273r		GAT AAC CAG TGT AAA GCT GT		
CSRICK5		GCC GCA ATG TCT TAT AAA TAT TCT	364	Raoult D., La Scola B., Enea M., *et al.* (2001) *Emerg Infect Dis* 7(1):73-81 [51]
CSRICK8		CCT TAG CTT TAG CTA TAT ATT T		
Rf190.1fw	*omp*A	ATG GCG AAT ATT TCT CTA AAA TTA	1860	Zavala-Castro J.E., Small M., Keng C., *et al.* (2005) *Am J Trop Meg Hyg* 73(4):662-666 [77]
Rf190.1800rev		TTA ACT CAC CAC CAC CGT TAG CAA GAC CG		
Rf190.1790fw		GTC TAC AGA TGA TAG AGT TAT CAC	1064	Pornwiroon W., Pourciau S.S., Foil L.D., *et al.* (2006) *Appl Environ Microbiol* 72(8):5589-5595 [55]
Rf190.2857rev		GTT TAA CTT CAG AGC CTG ACC G		
BM59	*omp*B	CCG CAG GGT TGG TAA CTG C	862	Roux V. and Raoult D. (2000) *Int J Sys Evol Microbiol* 50(4):1449-1455 [78]
B807		CCT TTT AGA TTA CCG CCT AA		
B607		AAT ATC GGT GAC GGT CAA GG	890	
B1497		CCT ATA TCG CCG GTA ATT		

120-2788f		AAA CAA TAA TCA AGG TAC TGT	812	
120-3599r		TAC TTC CGG TTA CAG CAA AGT		
120H-4108f		TCG GTG CTG CTG TCG GTA TCA	725	
120-4879r		TTA GAA GTT TAC ACG GAC TTT T		
RPOPULM25	*rpo*B	GTA ATT TTA TCA GTC AGG AG	564	La Scola B., Meconi S., Fenollar F., *et al.* (2002) *Int J Sys Evol Microbiol* 52(6):2035-2041 [52]
RPOPUL539		TGG ACT TTT CCT TCA TCA TG		
Bap355D		GAG CAA GAA GTA TAT ATG GG	1838	La Scola B., Meconi S., Fenollar F., *et al.* (2002) *Int J Sys Evol Microbiol* 52(6):2035-2041 [52]<br>Drancourt M. and Raoult D. (1999) *Antimicrob Agents Chemother* 43(10):2400-2403 [79]
RPOPUL2237		TCT ACC TGC TCA ACA ATA CC		
RPOPUL1939		CAA GGA GAG TTT ATT AAT TGC CG	1979	La Scola B., Meconi S., Fenollar F., *et al.* (2002) *Int J Sys Evol Microbiol* 52(6):2035-2041 [52]<br>Drancourt M. and Raoult D. (1999) *Antimicrob Agents Chemother* 43(10):2400-2403 [79]
Bap3850r		GCC CAA CAT TCC ATT TCD CC		
MQ32	*rff*E	TAA TCT TCT CAA CAC ACC CAA GGA C	358	Valarikova J., Sekeyova Z., Skultety L., *et al.* (2016) *Acta Virologica* 60(2):206-210 [71]
MQ33		GCT GCA GCA TAA TCC GAC AC		
pRFa	pRF plasmid	CAA GCT TTT GTA CTG CCT CTA T	159	Fournier P.E., Belghazi L., Robert C., *et al.* (2008) *PLoS One* 3(5):e2289 [81]
pRFb		AGT GCA TAT AGC TAC CAC ACT ATC T		
pRFc		ACA TTC CGT AAA GAA TAT GAG C	1342	
pRFd		GCT TAT GTT CGC CTT TAG TAT TTA		

* according to the *R. felis* reference strain URRWXCal2 sequence in GenBank (CP000053)

Table S3. Sequence similarities of selected rickettsial gene fragments of the Slovak isolate Danube with other *R. felis* strains (number of identical nucleotides/total with available sequences in GenBank).

	**Gene:**	***rrs***	***glt*A**			***sca*4**	***omp*A**		***omp*B**			***htrA***	***rpo*B**	***rff*E**
	Accession number	#ON053300	#ON053296	#ON053298	#ON053297	#ON053304	#ON053299	#ON053306	#ON053301	#ON053302	#ON053303	#ON053295	#ON053305	#ON053294
Culture isolates of *R. felis*	**URRWXCal2**<br>(#CP000053)	100%<br>1345/1345	100%<br>350/350	100%<br>318/318	100%<br>439/439	100%<br>1720/1720	100%<br>1721/1721	100%<br>1012/1012	100%<br>1518/1518	100%<br>770/770	100%<br>682/682	100%<br>499/499	100%<br>3868/3868	100%<br>313/313
	**LSU**<br>(#JSEM01000020)	100%<br>942/942	100%<br>350/350	100%<br>318/318	100%<br>439/439	100%<br>1720/1720	100%<br>1721/1721	100%<br>1012/1012	100%<br>1518/1518	100%<br>770/770	100%<br>682/682	100%<br>499/499	100%<br>3868/3868	100%<br>313/313
	**Pedreira**<br>(#LANQ01000001)	100%<br>1345/1345	100%<br>350/350	100%<br>318/318	100%<br>439/439	100%<br>1720/1720	100%<br>1721/1721	100%<br>1012/1012	100%<br>1518/1518	100%<br>770/770	100%<br>682/682	100%<br>499/499	100%<br>3868/3868	100%<br>313/313
	**CfCR(SJ)**	n/a	100%<br>350/350<br>JF694092	n/a	n/a	n/a	100%<br>380/380<br>JF694093	n/a	100%<br>244/244<br>JF694094	n/a	n/a	n/a	n/a	n/a
	**LSU-Lb**<br>(#JSEL01000013)	**99%**<br>**1343/1345**	100%<br>350/350	100%<br>318/318	100%<br>439/439	100%<br>1720/1720	100%<br>1721/1721	100%<br>1012/1012	**99%**<br>**1517/1518**	100%<br>770/770	100%<br>682/682	100%<br>499/499	100%<br>3868/3868	**99%**<br>**312/313**
